# BEYOND BORDERS: LONG-DISTANCE CAREGIVING AND SLEEP HABITS IN SOUTH ASIAN COMMUNITIES

**DOI:** 10.1093/geroni/igae098.3118

**Published:** 2024-12-31

**Authors:** Aziza Siddiqui, Srujana Chekuri, Akankshya Chataut, Dinesh Karki, Julie Blaskewicz Boron

**Affiliations:** University of Nebraska Omaha, Omaha, Nebraska, United States; University of Nebraska Omaha, Omaha, Nebraska, United States; University of Nebraska Omaha, Omaha, Nebraska, United States; University of Nebraska Omaha, Omaha, Nebraska, United States; University of Nebraska Omaha, Omaha, Nebraska, United States

## Abstract

Caregiving is generally altruistic in South Asian (SA) communities; however, caregiving activities can still create significant strain for caregivers depending on situational factors, including sleep and built environments (Hossain et al., 2020). Particularly, long-distance caregivers may grapple with the added complexities of not being physically present to provide care. This study utilized an online survey to investigate sleep habits and routines, caregiving activities, neighborhood satisfaction, and access to community resources for the SA community located in Omaha, Nebraska (N=74, MAge=25.8, SD=4.58). Caregivers reported on their perceived burdens, highlighting the unique challenges faced as they logistically navigate from afar, which remains underrepresented in research for the SA community (Hossein et al., 2020). Results revealed that SA caregivers’ sleep satisfaction was significantly related to caregiver responsibilities, including familial and professional expectations (χ2(6, N=74)=20.74,p<.05). Caregivers concerned about their sleep patterns were significantly more likely to have impaired quality of life (r(72)=0.62, p<.001). Self-rated home cleanliness was also considerable in caregivers’ controlling their bedtime (r(72)=0.36, p<.001). Thus, control over sleep length and quality are essential factors to reduce caregiver burden. This highlights that how better sleep habits and a clean surrounding environment can potentially reduce strain experienced by many long-distance caregivers. The impact of home environments for the long-distance SA caregiving community is yet to be fully understood. It would be valuable to investigate this community’s caregiver norms and cultural expectations further to promote environments conducive to sleep quality and overall quality of life.

